# Reproductive performance of pigs raised by intensive management system in Abuja, Nigeria

**DOI:** 10.14202/vetworld.2019.305-308

**Published:** 2019-02-22

**Authors:** Kenneth Owoicho Abah, Joy Iyojo Itodo, Simon Azubuike Ubah, Ibrahim Shettima

**Affiliations:** 1 Department of Theriogenology, Faculty of Veterinary Medicine, University of Abuja, Nigeria; 2 Department of Animal Science, Faculty of Agriculture, Federal University Kashere, Gombe State, Nigeria

**Keywords:** Abuja, intensive management system, reproductive performance, swine production

## Abstract

**Background:**

Population growth led to an increase in the number of people raising pigs, resulting in increased demand for piglets/pigs for breeding and pork for consumption.

**Aim:**

This study was carried out to determine the reproductive performance of pigs raised by the intensive management system in Abuja, Nigeria, with a view to assist farmers in ensuring improved productivity and profitability.

**Materials and Methods:**

Using an interview-based questionnaire, data from 121 sows and 649 preweaning piglets were collected in 12 herds, from September 2017 to March 2018. Measures of reproductive and production performance assessed in this study were interfarrowing interval (IFI), number of liveborn piglets (NLB), preweaning piglet mortality (PPM), age at weaning (AAW), weaning to service interval (WSI), age at first farrowing (AFF), number of piglets weaned per litter (NPWL), and number of piglets weaned per sow per year (NPWPY).

**Results:**

The results obtained in this study were IFI 6.2±0.84 months, NLB 7.2±1.11, PPM 31%, AAW 40.2±3.12 days, NPWL 5.3±0.73, WSI 39.4±4.59 days, AFF 9.1±0.60 months, and NPWPY 8.1±1.21. The identified causes of PPM were maternal overlay 31.34%, splay leg/hypoglycemia 22.39%, cannibalism 20.40%, starvation 14.93%, and unknown cause 10.94%.

**Conclusion:**

The result showed that the reproductive performance of the sow (especially, NPWPY and PPM) needs to be improved on. There is a need to promote extension and herd health services by veterinarians and livestock personnel to potential and existing farmers in the area. This is more so because organized pig production in the studied area is relatively new and more people are establishing pig farms in the studied area.

## Introduction

In some parts of Nigeria, swine production is an important source of income to livestock farmers. Pigs are raised mainly for sale, but there are other reasons why it is kept. This include festivals [1], home consumption, and for financial security [2-4].

Measures of production and reproductive performance include number of liveborn piglets (NLB), litter size at weaning, age at weaning (AAW), preweaning mortality, number of piglets weaned per sow per year (NPWPY), interfarrowing interval (IFI), farrowing rate, age at first farrowing, non-productive days, and weaning to conception interval [5-10]. Other indices used include average birth weight, average weaning weight, age of regular slaughter hogs sold, and average weight of regular slaughter hogs sold [10]. In breeding herds, the number of pigs weaned per sow per year is (from economic point of view) the most important measure of productivity [1].

Although some researchers studied the production and reproductive performance of pigs in few towns and cities in Nigeria [1,3,4,10], similar work has not been reported among pigs raised in Abuja and its environs. Such information is important because when the productivity and profitability of smallholder piggeries are increased, social and economic well-being of pig farmers in the study area will improve [2].

This study aimed to obtain information on pig production in smallholder piggeries: Their reproductive performance and production characteristics.

## Materials and Methods

### Ethical approval

All applicable international, national, and/or institutional guidelines for the care and use of animals were duly followed.

### Informed consent

The consent of the swine farmers was sort before the commencement of the interview. Only people who gave approval for an interview were included in the study.

### Study area

This study was carried out in Abuja. Abuja is the capital city of Nigeria. It is located in the center of Nigeria within the Federal Capital Territory (FCT). It has a land area of about 8000 square km. Abuja lies at latitude 9.07°N and longitude 7.48°E and an elevation of 840 m (2760 ft) above sea level. It is bounded on the north by Kaduna State, on the west by Niger State, on the east and southeast by Nasarawa State, and on the southwest by Kogi State [11]. The FCT is divided into six area councils, namely Abuja municipal, Gwagwalada, Abaji, Kuje, Bwari, and Kwali area councils.

### Selection and recruitment of pig farms and pig raisers

A snowball sampling procedure was used to select pig farms and pig raisers for the study. A total of 12 commercial and intensively managed pig farms were selected based on the willingness of the owners to make their farms and farm records available to the researchers.

### Data collection

The selected herds were visited once a month by the investigator from April to October 2017. Data on sow reproductive performance and production characteristics were collected using a pre-tested interview-based questionnaire. The questionnaire covered issues related to the manager (the person who takes care of the farm), owner, individual sows, general herd reproductive factors, general piglet factors, herd facilities, general herd nutrition, general issues of herd hygiene and disease control, and culling.

During the monthly visits, sows and piglets were monitored, and morbidity and mortality data were recorded. Reproductive problems of the sows were also monitored, and piglet mortality was recorded during the visits.

### Measures of sow reproductive performance

Eight animal- and herd-level measures of sow reproductive performance were used in this study: IFI (interval between the last and second last farrowing in months), NLB at the last recorded farrowing, preweaning piglet mortality (PPM) (percentage at the last farrowing), AAW, weaning to service interval (WSI), NPWL, NPWPY, and AAW.

### Measures of health in preweaning pigs

Measures of health in preweaning pigs were determined by recording the causes of mortality. Mortality was determined by recording all cases of death in pigs.

### Statistical analysis

The data were entered into the Excel datasheet, and descriptive statistics were performed. The values for IFI, NLB, PPM, AAW, WSI, NPWL, NPWPY, and AAW were expressed as mean±standard deviation. Similarly, stillbirth and PPM at sow and herd level were expressed as percentages.

## Results

### General information

During the study, it was observed that almost all the farm owners are residents of Abuja municipal, but the farms are located in the outskirt and satellite towns. The main reason was the high cost of land in the city, overpopulation, and high concentration of residential houses.

In total, 12 medium holder commercial pig farms were involved in the study: Farms A, B, and C in Kubwa, D and E in Old Wuse, F, G, and H in Masaka, I and J in Zuba, and K and L in Uke. In total, 121 sows were included in the study.

### Sow reproductive performance

Table-1 shows the reproductive performance parameters of sow herds in Abuja and its environs. Herd B presented the worst performance. Sows in this herd had not littered live piglets for 24 months. There was a normal reproductive cycle; hence, WSI was normal. The sows had repeated abortions and stillbirth. For all the herds, IFI (6 months), PPM (31%), and NPWPY (8) were achieved. Disparities in some performance indices are wide among the herds.

**Table-1:** Reproductive performance parameters of sow herds in Abuja (herds A-L).

Parameter	A	B	C	D	E	F	G	H	I	J	K	L	Total
IFI	5	5	NA	NA	7	NA	12	6	5	6	NA	5	6.25
NLB	10	0	11	7	4	13	6	8	11	7	NA	6	7.50
PPM (%)	27	100	13.5	30	20	27	40	15	NA	12	NA	30	31.79
AAW (days)	30	56	30	42	42	30	30	42	56	56	30	42	39.83
NPWL	6	0	9.2	4	3	7	3	6	4	5	NA	5	5.00
WSI (days)	30	30	NA	60	NA	60	30	50	40	30	NA	40	39.01
Age at first farrowing (month)	7	12	12	11	9	NA	8	8	11	NA	9	9	9.08
NPWPY	11	0	14	5	4	12	6	12	7	9	NA	8	8.01

IFI=Interfarrowing interval, NLB=Number of liveborn piglet, PPM=Preweaning piglet mortality, AAW=Age at weaning, NPWL=Number of piglets weaned per litter, WSI=Weaning to service interval, NPWPY=Number of piglets weaned per sow per year

### Measures of health in preweaning pigs

Table-2 shows the identified causes of PPM in pig farms in Abuja and its environs. Preweaning mortality occurs mostly during the 1^st^ week postpartum. The most frequent cause of death is a maternal overlay (31.34%) followed by splay leg/hypoglycemia (22.39%) (Table-2).

**Table-2:** Identified causes of PPM in pig farms in Abuja.

Cause	Number	Mortality (%)
Maternal overlay	63	31.34
Splay leg/hypoglycemia (a)	45	22.39
Cannibalism (b)	41	20.40
Starvation	30	14.93
Unknown	22	10.94
Total	201	100

(a) The piglets were born with extended hind legs. This prevents them from walking and approaching the nipple to suckle.

(b) In some herds, pregnant sows were sometimes kept together with other pigs. These resulted in the high incidence of cannibalism, PPM=Preweaning piglet mortality

## Discussion

Pig production in Abuja and its environs is a reflection of the multiethnic nature of the city as piggery owners came from different parts of Nigeria, mostly areas where pork consumption is not considered a taboo. Farmers buy land and build piggeries where the animals are kept. Large white and landrace are the most common breeds kept in Abuja and are the breeds used in this study. Native pigs/crosses are kept under an extensive system of management by natives/locals in only a few places such as Giri, Anguwan Dodo, and Dobi all in Gwagwalada Area Council.

The reproductive performance of sows in this survey was relatively low as compared to commercial piggeries in temperate and tropical regions including Nigeria [1,4,12-15]. On average, they are also low compared to results obtained from smallholder studies elsewhere [3,4].

The IFI (6.2 months) is higher than the value (4.7 months) reported in institutional herds in Benue [1]. The difference may be attributed to better management practice in the institutional herds. However, the IFI observed was similar to the one observed in smallholder piggeries in other tropical regions [3,4]. Lanada *et al*. [2] reported that certain factors such as a number of non-productive days, gestation length, and lactation length are responsible for prolonged IFI.

The NPWPY was 8.0. This value is very low compared to the figure (15.0) reported for institutional herds in Benue State [1]. It is lower than the figure (10.5) considered as a good performance [16]. The value is also lower than figure (10.7) reported for smallholder piggeries in Northern Nigeria [3].

PPM (31%) recorded in this study is higher than the acceptable value of 10% for pigs [1]. This value is very bigger than 20%, which lambs preweaning mortality [17]. From Table-2, the most frequent cause of piglet mortality (31%) in the study area is a maternal overlay, followed by splay leg/hypoglycemia, cannibalism, starvation, and unknown causes, respectively. Similar to Çilek [17], it was observed that the high preweaning mortality could be attributed to poor management practice and inadequate feed given to the animals. As sometimes, certain feedstuff that provides energy and protein for the pregnant sows is not included in the ration. The value (31%) is higher than the figure (17.75%) reported for pigs in Nsukka, Enugu State, Nigeria [10]. The lower figure obtained in this study may be due to the higher ability of crossbred piglets to resist endemic diseases than exotic breeds. The value is lower than 50% reported for native pigs in smallholder production systems in Northern Lao PDR [18]. This may be as a result of poor hygiene and lack of disease preventive measures during gestation and lactation observed in the study at Northern Lao PDR.

Around 50% of PPM is caused by maternal overlying [6]. Farrowing management contributes to the number of mortality as sows kept in a farrowing crate are reported to be the most secure way of reducing this mortality [10]. The killing or injury to piglets (cannibalism) by the dam represents a significant source of piglet mortality. In Nsukka, Southeast Nigeria, the value obtained this study (20%) is higher than the report by Abonyi *et al*. [10]. The reason is the same as was mentioned earlier for the likely factors responsible for high piglet mortality in the study area.

From Table-1, Herd B presented the worst performance. Sows in this herd had not littered live piglets for 24 months. There was a normal reproductive cycle; hence, WSI was normal. The sows had repeated abortions and stillbirth. The researchers are trying to investigate the cause(s) of infertility. Feed sample from the farm was collected for proximate analysis to determine the nutritional content and presence of toxins in the feed. Blood sample and vaginal swab from sows that aborted were collected for the screening of brucellosis. The blood sample will also be used for hormonal analysis to determine whether the problem is caused by hormonal imbalance.

NLB in this study was 7.0. This value compares with that reported by Ate and Oyedipe and Abonyi *et al*. [1,10]. Their figures are 7.45 and 7.5, respectively. It is lower than the values reported for commercial farms in many temperate countries [16,19-21] and smallholder farms in Western Kenya and the Philippines [2,15]. Lanada *et al*. [2] reported that the nutrition of sows around the time of conception influences litter size in the commercial industry. This may explain why the NLB is low in our study as the nutritional intake of sows (both in terms of quality and quantity) is generally poor. Only farm C occasionally adds the commercial feed to the sow diet.

We observed that pigs are routinely treated for external and internal parasites once every 2-4 months by people who may not have been doing it well (non-vets). Most of the farms use to wash/clean the pens every morning and disinfect the pen using Izal once a week. Estrus detection methods are employed and well known by the farm managers. Most of the methods used include observation of reddish and swollen vulva, restlessness, and whitish vulval discharge 3 weeks after farrowing.

Feeding in the farms in this locality follows a similar pattern. Homemade concentrates were fed the pigs in an unspecified amount, 2 or 3 times a day. Creep feeding is not practiced as both the piglets and adults are served the same feed. Items mixed include maize bran, palm kernel cake, brewers waste, and wheat offal. Occasionally, soybeans, bone meal, methionine, lysine, toxin binders, and premixes are added to the ration. Piglets are injected with iron dextran 3 days after birth. About 41% of the farms wean their piglets at 30 days. The mean AAW is 40 days. We are recommending that farmers wean their litters as soon as possible after 30 days of age. If creep feeding is introduced (which we are recommending), litters can be weaned at 21 days of age, as early weaning is associated with reduced IFI [2].

## Conclusion

The reproductive performance of the sow (especially, NPWPY and PPM) is very poor. There is a need to promote extension and herd health services by veterinarians and livestock personnel to potential and existing farmers in the area. This is more so because organized pig production in the studied area is relatively new and more people are establishing pig farms in the area.

## Authors’ contributions

KOA designed the study, wrote the protocol, wrote first draft of the study, and performed the field work. JII, SAU, and IS managed the analyses and literature searches and participated in data collection. All authors read and approved the final manuscript.
